# Peanut and Soy Protein-Based Emulsion Gels Loaded with Curcumin as a New Fat Substitute in Sausages: A Comparative Study

**DOI:** 10.3390/gels11010062

**Published:** 2025-01-13

**Authors:** Hong-Yan Yan, Shao-Bing Zhang

**Affiliations:** College of Food Science and Engineering, Henan University of Technology, Zhengzhou 450001, China; yanhongyanz@163.com

**Keywords:** sausages, emulsion gels, peanut proteins, soy proteins, ultrasound modification, curcumin

## Abstract

The aim of this study was to evaluate the effects of the complete or partial substitution (0, 20, 40, and 100%) of the pork backfat in prepared sausage with protein emulsion gels loaded with curcumin. The effects of three protein emulsion gels (i.e., peanut proteins, ultrasound-modified peanut proteins, and soy proteins) on sausage characteristics (cooking loss, textural properties, microstructure, sensory characteristics, and antioxidant activity) were investigated and compared using a one-way analysis of variance and Duncan’s multiple tests. The results revealed that the addition of each emulsion gel reduced cooking loss and improved the textural properties of the sausages in a dose-dependent manner. When 20% of pork backfat was substituted with untreated or ultrasound-modified peanut protein emulsion gel (PPEG), cooking loss decreased to a greater extent than when soy protein emulsion gel (SPEG) was used. However, the latter yielded higher cohesiveness and resilience at the same substitution levels. Compared with untreated PPEG, the sausages containing modified PPEG (at 200 W for 20 min) had significantly greater resilience and a denser microstructure. In addition, when 100% of pork backfat was substituted with modified PPEG, the sausages had desirable sensory characteristics. All sausages enriched with protein emulsion gels loaded with curcumin presented higher DPPH and ABTS radical scavenging capacities than the control sausages. The sausages prepared with the modified PPEG had the highest antioxidant activity (DPPH: 37.43 ± 0.35%; ABTS: 39.48 ± 0.50%; TBARS: 0.65 ± 0.05 mg MDA/Kg), which may be attributed to the increased stability of curcumin in the modified PPEG with a denser network structure. Therefore, ultrasound-modified PPEG loaded with curcumin can be used as a new fat substitute in functional sausages or other healthy meat products.

## 1. Introduction

Animal fats contain high levels of saturated fatty acids (SFAs) and cholesterol, which may increase the incidence of cardiovascular disease, obesity, and cancer [1]. As a result, healthy meat products that are low in animal fat are increasingly favored by consumers. However, directly reducing the fat content in the formula may cause undesirable effects, including weakness of taste and texture and lower acceptability of products. A previous study suggested that plastic fat can be created by structuring vegetable oils in liquid form, which retain solid-like properties while having a healthier fatty acid profile and avoiding undesirable quality changes in the final, reformulated meat products [2].

An emulsion gel is a semisolid gel material with a three-dimensional network structure formed by emulsifying liquid vegetable oils into a gel matrix through certain induction methods [3]. Several studies [4,5,6] have shown that meat products made with emulsion gels as fat substitutes not only maintain their hardness, flavor, and acceptability but also have an improved fatty acid composition. In recent years, there has been an increase in research on the use of emulsion gels loaded with bioactive compounds to prepare functional meat products. Pintado, Munoz-Gonzalez, Salvador, Ruiz-Capillas, and Herrero [7] reported that frankfurters with emulsion gels containing solid polyphenol extracts (from either grapeseed or grapeseed and olive) presented high levels of hydroxytyrosol and high contents of gallic acid, flavanol monomers, and their derivatives, and also that these nutritional advantages did not lead to any undesirable sensory, structural, or stability changes. Curcumin is a naturally occurring polyphenolic compound extracted from the turmeric plant, which has attracted attention for its positive physiological effects, such as its antioxidant, anti-atherosclerosis, anti-cancer and anti-inflammatory activities [8]. Li et al. [9] employed zein/carboxymethyl dextrin Pickering emulsion gels loaded with curcumin as a fat substitute in sausages and reported that the addition of curcumin further reduced cooking loss and improved the oxidative stability of sausages.

Plant proteins are ideal materials for preparing protein emulsion gels. Currently, emulsion gels prepared from soy proteins have been widely used in the development of healthier meat products [10,11,12], but there are few reports on the application of peanut protein emulsion gels, which may be related to some of the weaker functional properties of the latter. In fact, peanut proteins have better flavor and fewer antinutritional factors than soy proteins. In addition, China is the world’s largest producer of peanuts, and peanut proteins are readily available. If the functional properties of peanut proteins are improved by structural modification, they could have greater potential for application in food processing. Ultrasound modification is a physical technique with mechanical and cavitation effects that can cause subunit dissociation or aggregation, break peptide bonds, and alter the structural and functional characteristics of food proteins [13]. Our previous research revealed that emulsion gels prepared from peanut proteins treated with medium-intensity ultrasound (200 W/20 min) had stronger textural properties. Moreover, ultrasound significantly improved the storage stability, photostability, and bioavailability of curcumin in emulsion gels [14]. Therefore, it was hypothesized that ultrasound-modified peanut protein emulsion gels could be applied in the preparation of functional sausages and improve sausage quality, such as healthiness, sensory qualities, and shelf life.

The objective of this work was to evaluate the effects of the partial or complete substitution of pork backfat in prepared sausage with three types of protein emulsion gels (i.e., peanut proteins, ultrasound-modified peanut proteins, and soy proteins) loaded with curcumin. The effects of protein emulsion gels on the cooking loss, textural properties, microstructure, and sensory properties of sausages at different fat substitution levels (20, 40, and 100%) were investigated; subsequently, the antioxidant activity (DPPH and ABTS radical scavenging and the inhibition of lipid oxidation) of the functional sausages was further evaluated. This work expands the application of peanut proteins in the food industry and provides a theoretical and practical basis for the development of healthy meat products.

## 2. Results and Discussion

### 2.1. Cooking Characteristics

**Figure 1:**
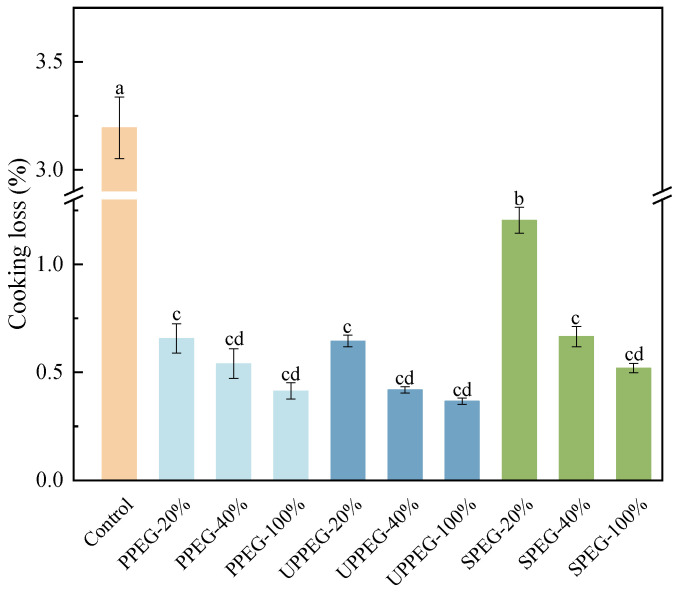
Cooking loss from sausages after replacing pork backfat with different protein emulsion gels. Control: without emulsion gel; PPEG: peanut protein emulsion gel; UPPEG: ultrasound-modified peanut protein emulsion gel; SPEG: soy protein emulsion gel. Values are mean ± standard error (*n* = 3). Different letters indicate significant differences (*p* < 0.05).

Cooking loss is an important index used to judge the quality of meat products [15]. As shown in Figure 1, compared with those of the control sausages, the cooking loss rates of sausages containing added emulsion gel were reduced to varying degrees. This decrease in cooking loss was attributed mainly to the network structure formed by the interaction of emulsion gel particles with the meat paste so that the sausages could retain the juices better [16]. As the amount of emulsion gel increased, the cooking loss rate of the sausages tended to decrease. This may be because the increase in protein content enhanced protein interactions, resulting in the formation of a denser network structure with an improved ability to bind water and other components, thereby reducing the cooking loss rate [17]. Compared with soy protein emulsion gel (SPEG), untreated or ultrasound-modified peanut protein emulsion gel (PPEG) decreased cooking loss to a greater extent at a 20% substitution level, but it was not significantly different at a 40% and 100% substitution level. From the perspective of reducing cooking losses only, compared to SPEG, the application of PPEG in sausages at a 20% fat substitution level was most effective, suggesting that peanut proteins may have stronger interactions with the meat paste.

**Figure 2:**
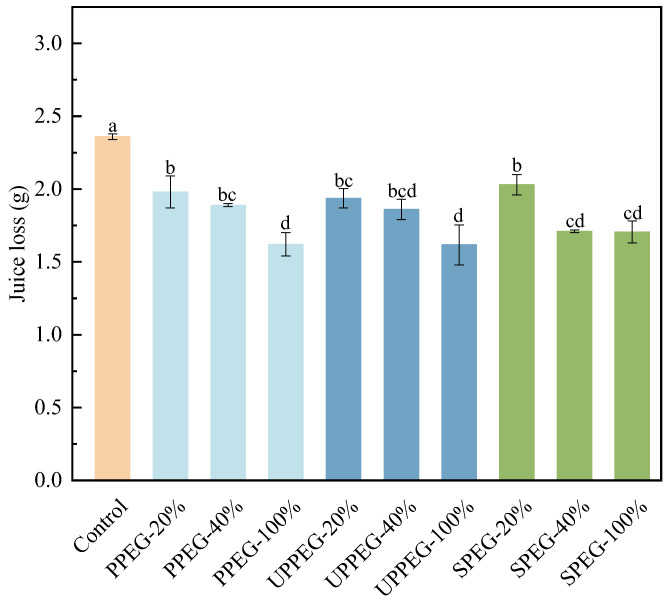
Juice loss from sausages after replacing pork backfat with different protein emulsion gels. Control: without emulsion gel; PPEG: peanut protein emulsion gel; UPPEG: ultrasound-modified peanut protein emulsion gel; SPEG: soy protein emulsion gel. Values are mean ± standard error (*n* = 3). Different letters indicate significant differences (*p* < 0.05).

After cooking, the fresh sausages were vacuum-packed and stored at 4 °C overnight and then juice loss was evaluated. As shown in Figure 2, juice loss was significantly reduced in all the sausages due to the addition of protein emulsion gels. The amount of juice lost decreased with an increasing proportion of emulsion gel; this may be because emulsion gels help sausages form a more effective water-holding network structure. Our previous research [18] reported that all three types of protein emulsion gels had excellent water-holding capacity (≥78%). There were no significant differences in juice loss among sausages prepared with the three types of protein emulsion gels at different substitution levels. Paglarini, Martini, et al. [19] reported that sausages containing emulsion gels had better emulsifying stability and lower juice loss. Therefore, replacing animal fat with emulsion gel in sausages can provide a stable emulsified meat paste system, thereby preventing juices from seeping out of the sausages during storage.

### 2.2. Texture Profile of Sausages

Hardness is defined as the force required for the deformation of an object and springiness is defined as the ability of an object to regain its original size and shape after deformation. As shown in Table 1, the textural properties (hardness and springiness) of the sausages were significantly improved after emulsion gel addition compared with the control sausages; this may be caused by the different physical states of pork backfat and emulsion gel in the sausages. Since the pork backfat was minced, its tissue structure was destroyed and had collapsed. In contrast, the microgel particles were able to reaggregate and form a network structure during sausage production, resulting in the stronger textural properties of the fat-substituted sausage. Ozturk-Kerimoglu, Kavusan, Benzer Gurel, Cagindi, and Serdaroglu [20] used cold-set emulsion gels prepared with albumin (hen egg white) and a mixture of oils (cold-pressed peanut oil and linseed oil) as a fat substitute in fermented beef sausages and reported that all sausages containing cold-set emulsion gels presented increased hardness. In addition, the textural strength of the sausages increased further with increasing emulsion gel substitution; this can mainly be attributed to the semisolid nature and good network formation capability of protein emulsion gels. Therefore, in the subsequent experiments, high emulsion gel substitution levels (40% and 100%) were selected for the preparation of sausages.

The springiness and hardness of all sausages at the same levels of fat substitution were not significantly different (Table 1). However, the sausages prepared with SPEG presented greater cohesiveness at a 40% fat substitution level. Resilience is one of the key indicators reflecting the internal textural strength of food. At a 20% fat substitution level, the sausages containing ultrasound-modified PPEG had significantly greater resilience than those prepared with untreated PPEG. This increase in the resilience of the sausage may be attributed to the high elastic modulus (G’) of the ultrasound-modified PPEG, as reported in our previous work [14]. Paglarini, Furtado, et al. [21] prepared functional emulsion gels containing chia seed powder based on SPI and inulin, which were used to replace pork backfat. They reported that emulsion gels with high G’ values contributed greatly to the stronger textural properties of the sausages.

### 2.3. Appearance and Microstructure of Sausages

**Figure 3:**
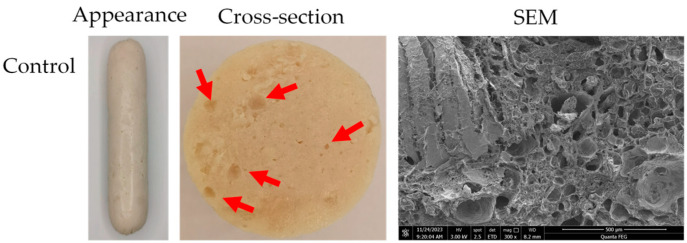

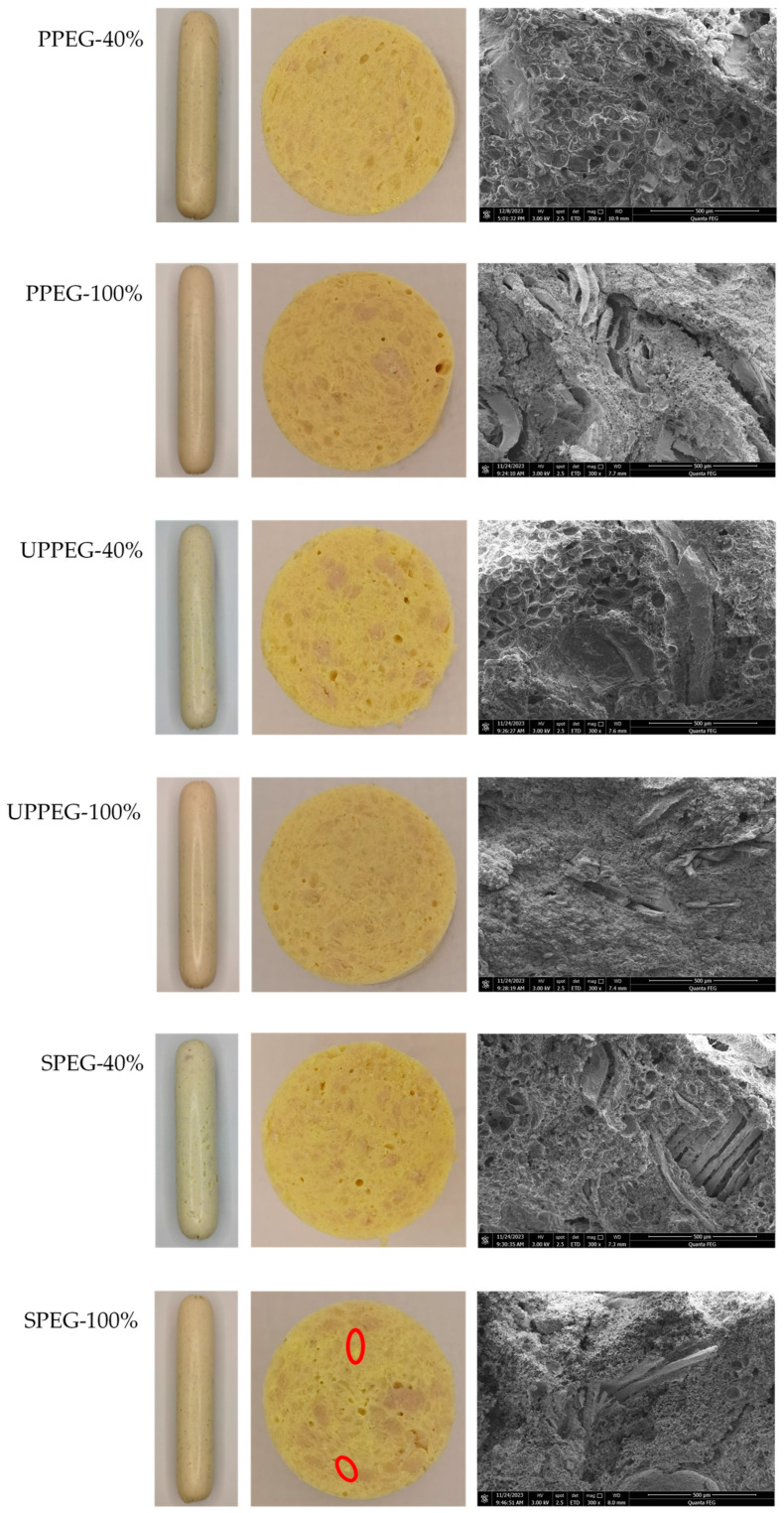
Appearance, cross-sections, and SEM images of sausages after replacing pork backfat with different protein emulsion gels. Control: without emulsion gel; PPEG: peanut protein emulsion gel; UPPEG: ultrasound-modified peanut protein emulsion gel; SPEG: soy protein emulsion gel. SEM images represent cross-sections of freeze-dried sausages. The bars in the SEM images indicate a length of 500 μm. Holes and gel clumps in the surface (cross-section) are indicated by red arrows and circles, respectively.

As shown in Figure 3, all sausages had a similar appearance. The cross-sections of the sausages in the control group showed visible pores and were not dense enough, which indicated a poor internal structure. The cross-sections of the sausages containing added SPEG had milky white gel clumps, possibly because the SPEG was not easy to churn uniformly.

**Figure 4:**
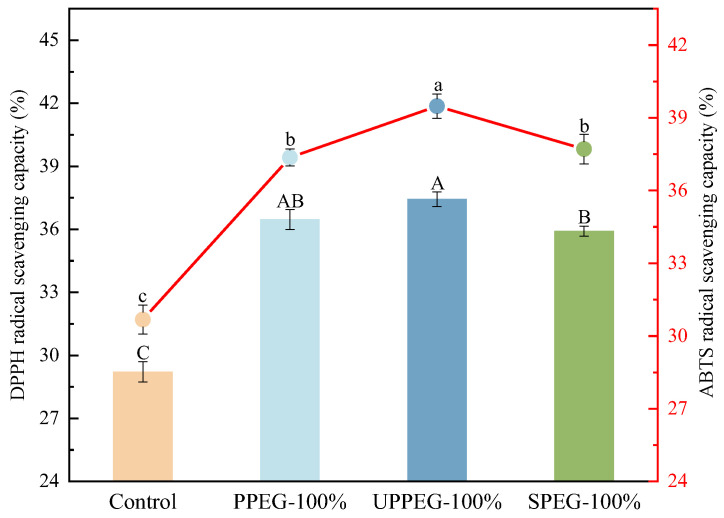
Radical scavenging capacity of freshly cooked sausages with different protein emulsion gels. Control: without emulsion gel; PPEG: peanut protein emulsion gel; UPPEG: ultrasound-modified peanut protein emulsion gel; SPEG: soy protein emulsion gel. Values are mean ± standard error (*n* = 3). Different letters indicate significant differences (*p* < 0.05).

Furthermore, as shown by the SEM images of the sausages, the sausages in the control group presented a honeycomb-like structure with an uneven cavity size distribution, which is consistent with the findings of Ferrari Felisberto et al. [22]. The formation of these holes may have been due to the expansion of a number of constituents, such as fat, water, air, or starch [23]. The three-dimensional network structures of the sausages became uniform with increasing emulsion gel concentration. When the percentage of fat substituted with emulsion gel was 100%, all the sausages were denser and more homogeneous (Groups C, E, and G in Figure 4), and the honeycomb structure disappeared; this could explain why these sausages presented the lowest cooking losses (Figure 1) and stronger textural characteristics (Table 1). Compared with the untreated PPEG (Group C) and SPEG (Group G) sausages, the microstructure of the sausage containing ultrasound-modified PPEG (Group E) was denser and more homogeneous, suggesting that the latter has greater potential to improve the internal structure of sausages; this may be attributed to the increase in exposed hydrophobic and free sulfhydryl groups of peanut proteins after ultrasound modification, which leads to enhanced protein–protein interactions [24].

### 2.4. Sensory Evaluation

As shown in Table 2, the sausages with added protein emulsion gels scored significantly higher than those in the control group in terms of texture and acceptability, but there was no significant difference between the two groups in terms of appearance, color, or flavor (*p* > 0.05). With increasing emulsion gel levels from 40% to 100%, only the sausages containing ultrasound-modified PPEG and SPEG showed significant improvements in acceptability. Among all the sausages with added emulsion gels, the sausages containing modified PPEG had higher scores of acceptability and texture. The results indicated that the use of protein emulsion gels to replace animal fat not only had nutritional advantages but also improved certain sensory characteristics of the sausages. Delgado-Pando, Cofrades, Ruiz-Capillas, and Jimenez-Colmenero [25] prepared emulsion gels using sodium caseinate-SPI and a mixture of oils (olive, linseed, and fish oils) as a substitute for pork backfat in low-fat frankfurters. They reported that the substitution of emulsion gels did not affect the sensory characteristics of the frankfurters.

### 2.5. Antioxidant Activity

The determination of DPPH and ABTS radical scavenging capacities is frequently used to evaluate the antioxidant activity of substances. As shown in Figure 4, the control sausages presented DPPH and ABTS radical scavenging capacities, which may be attributed to the radical scavenging capacity of certain components in the meat itself. However, the DPPH and ABTS radical scavenging capacities of the sausages containing emulsion gels were significantly greater than those of the control group, which was related to the strong radical scavenging capacity of the curcumin in the emulsion gels. Curcumin can readily transfer electrons or easily donate H atoms from two phenolic sites to scavenge free radicals [26]. Among the sausages prepared using three types of protein emulsion gels, those containing ultrasound-modified PPEG presented the highest DPPH and ABTS radical scavenging capacities. The differences in the antioxidant activities of these sausages may be attributed to the different amounts of curcumin. Although the initial curcumin contents in the raw sausages were identical, the curcumin content after cooking might vary significantly due to the different protective capacities of the emulsion gels. Our previous research revealed that the stability of curcumin in emulsion gels prepared from ultrasound-modified peanut proteins was better than that in untreated protein emulsion gels [14]. Liu et al. [27] used ultrasound-modified spirulina protein to prepare high-internal-phase emulsions loaded with curcumin and reported similar results.

**Figure 5:**
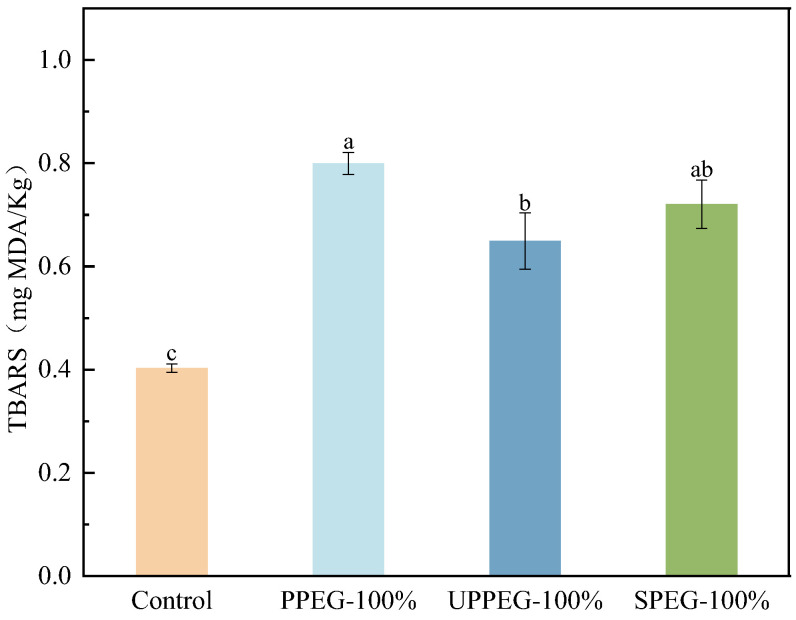
TBARS values for fresh cooked sausages with different protein emulsion gels. Control: without emulsion gel; PPEG: peanut protein emulsion gel; UPPEG: ultrasound-modified peanut protein emulsion gel; SPEG: soy protein emulsion gel. Values are mean ± standard error (*n* = 3). Different letters indicate significant differences (*p* < 0.05).

The TBARS value is an important indicator for evaluating the degree of fat oxidation in meat products and represents the content of malondialdehyde (MDA), a secondary oxidation product of fat. In general, TBARS values close to 0.5 mg/kg indicate the beginning of oxidation, whereas TBARS values higher than 1.0 mg/kg indicate more advanced oxidation [28]. Figure 5 shows that the TBARS values of all the fresh sausages were below the rancid limit (<1.0), indicating that all the sausages presented low levels of lipid oxidation. However, the TBARS values of the sausages containing emulsion gels were all significantly greater than those of the control group. This occurred because animal fats contain a large amount of saturated fatty acids, which are chemically stable, whereas the soybean oil in the emulsion gel contains a high proportion of unsaturated fatty acids, which are chemically active and prone to oxidation [29]. Therefore, the susceptibility of vegetable oils to oxidation may be the main disadvantage of using emulsion gels as a substitute for animal fats in sausage production.

Compared with the sausages containing untreated PPEG or SPEG, the sausages with modified PPEG had lower TBARS values; this may be because the latter contained more curcumin after cooking, as discussed above. Li et al. [9] prepared sausages by substituting pork backfat with carrageenan-zein/carboxymethyl dextrin emulsion gels loaded with curcumin and reported that the addition of curcumin further decreased lipid oxidation in the sausages, and also that the sausages exhibited the best oxidative stability at a 100% substitution level. Curcumin can effectively scavenge lipid free radicals to some extent [9,30], thereby delaying the oxidation of vegetable oil in sausages. The addition of curcumin not only enriched the nutrient value of the sausages but also played an antioxidant role, which was very effective in prolonging the shelf life of the products.

## 3. Conclusions

Substituting pork backfat with PPEG or SPEG in sausages could reduce their cooking loss and improve their textural properties. At the same substitution levels, sausages prepared with untreated PPEG had lower cooking loss but weaker textures than those prepared with SPEG. However, sausages containing ultrasound-modified PPEG had a denser and more homogeneous microstructure. When 100% pork backfat was substituted with modified PPEG, the sausages presented desirable sensory characteristics. In addition, the addition of protein emulsion gels loaded with curcumin significantly increased the DPPH and ABTS radical scavenging capacities of the sausages. Those sausages prepared with modified PPEG had the highest antioxidant activity. The results indicate that ultrasound-modified PPEG loaded with curcumin can be used as a new fat substitute in the development of functional sausages or other healthy meat products. However, there remains a need for future research into more effective means of controlling the oxidation of vegetable oils in emulsion gels, and the digestion and absorption behaviors of functional sausages require further investigation as well.

## 4. Materials and Methods

### 4.1. Materials

Peanuts were obtained from the local market. Soy protein isolates (YP928C) were purchased from Shandong Yuwang Ecological Food Industry Co., Ltd. (Yucheng, China). Lean pork and pig backfat were purchased from the Yonghui Supermarket Co., Ltd. (Zhengzhou, China). The 23 mm in diameter food-grade plastic casings (made of polylactam, polyethylene, and nylon; thickness, 0.1 mm) were purchased from Taobao. Soybean oil was purchased from the COFCO Yellow Sea Grain and Oil Industry Co., Ltd. (Rizhao, China). Gluconic–delta–lactone (GDL), 1,1-diphenyl-2-picrylhydrazyl (DPPH), 2-thiobarbituric acid (TBA), and 2,2′-azino-bis (3-ethylbenzothiazoline-6-sulfonic acid) diammonium salt (ABTS) were purchased from Shanghai McLean Biochemical Technology Co., Ltd. (Shanghai, China). Curcumin (≥95%), trichloroacetic acid, and potassium persulfate were purchased from Tianjin Kemio Chemical Reagent Co., Ltd. (Tianjin, China).

### 4.2. Extraction and Ultrasonic Treatment of Peanut Proteins

Peanut proteins were extracted according to the method used by Jiang, Zhang, Zhang, and Peng [31]. Peanut protein powder was mixed with distilled water and stirred overnight at room temperature to obtain a peanut protein solution (5.7%, *w*/*v*, pH 7.0). Afterward, peanut protein solution (35 mL) was taken into a 50 mL beaker for modification with an ultrasonic cell disintegrator (Sci-entz-IID, Ningbo Scientific Biotechnology Co., Ltd., Ningbo, China) at 200 W for 20 min (without temperature control). The final temperature of the peanut protein solution was 45 ± 2 °C.

### 4.3. Preparation of Protein Emulsion Gels Loaded with Curcumin

Curcumin was added to the soybean oil to achieve a final concentration of 0.03 g/mL, then the mixture was stirred at 60 °C under light protection for 2 h to obtain soybean oil dissolved with curcumin. Then, different proteins (peanut proteins, ultrasound-modified peanut proteins, and soy proteins) were dispersed in distilled water and stirred overnight at room temperature (pH 7) to obtain different protein solutions (4%, *w*/*v*), which were mixed with soybean oil (30%, *v*/*v*) dissolved with curcumin. The mixture was processed at 15,000 r/min for 2 min 20 s by a digital high-speed homogenizing and dispersing machine (FJ300-PSH, Shanghai Specimen Model Factory Co., Ltd., Shanghai, China), then sonicated (Scientz-IID, NingBo Scientz Biotechnology Co. Ltd., Ningbo, China) at 300 W for 20 min (pulse duration of 3 s and off time of 2 s, <35 °C) to obtain a fine protein emulsion loaded with curcumin [31]. Finally, GDL was added to peanut protein, ultrasound-modified peanut protein, and soy protein emulsions (20 mL) to achieve final concentrations of 0.2%, 0.3%, and 0.5% (*w*/*v*), respectively. The mixture was stirred until the GDL dissolved completely and then heated for 30 min in an 85 °C water bath to form a gel (4 variants). After heating, the gel was immediately cooled in ice-cold water and stored overnight at 4 °C.

### 4.4. Preparation of Functional Sausages

The formulations of sausages in which a proportion of pork backfat was replaced with different levels of protein emulsion gels are shown in Table 3. The lean meat and backfat were chopped into small pieces and then minced using a meat grinder. Afterward, the other components of the formulation were added to the minced meat and mixed well to obtain a meat paste. The meat paste (100 g) was poured into plastic casings 23 mm in diameter to obtain two raw sausages. The weight of a raw sausage was 40 g ± 2 g; the diameter was 23 mm, and the length was 13 cm ± 1 cm. Sausages were prepared in three separate batches on different days, with each batch including two sausages for each treatment.

### 4.5. Determination of the Cooking Characteristics of Sausages

#### 4.5.1. Cooking Loss

The raw sausages were cooked for 30 min at 85 °C in a water bath. Three independent batches of sausages (each batch included two sausages) for each treatment were cooked on different days. The cooking loss of the sausages was expressed as a percentage of the weight of sausage lost after heat processing, relative to the weight of the initial sample.

#### 4.5.2. Juice Loss After Storage

The cooked sausages (cooked at 85 °C for 30 min) were removed from their plastic casings and stored at 4 °C overnight (vacuum-packed). The juice loss after storage was expressed as the difference between the weight of the freshly cooked sausage and the weight of the sausage after storage overnight at 4 °C.

### 4.6. Texture Profile Determination

The texture of the sausages was determined according to Choi et al. [32], with some modifications. The cooked sausages (with the plastic casings removed) were stored overnight at 4 °C (under vacuum packaging). Afterward, the samples were removed and kept at room temperature for 1 h and then cut into cylinders of 20 mm × 23 mm (height × diameter). The hardness, springiness, cohesiveness, and resilience of the central portion of each sausage sample were determined via a texture analyzer (TA-XT Plus, Stable, UK) with a P/36 probe. The test speed was 1 mm/s and the compression ratio was 50%.

### 4.7. Scanning Electron Microscopy (SEM)

The sausages were cut into slices of approximately 1 mm in thickness; the cross-sections were photographed with a mobile phone (Huawei Honor V30), frozen at −20 °C for 12 h, and then freeze-dried for 24 h. The freeze-dried sausage slices were fixed on a copper plate, sprayed with gold, and then observed and photographed via SEM (Quanta 250FEG, FEI, Hillsboro, OR, USA).

### 4.8. Sensory Evaluation

To determine the sensory effects of emulsion gels loaded with curcumin on sausages, a sensory evaluation panel consisting of 15 laboratory personnel (4 men and 11 women, aged 24–26) was convened. The cooked sausages (at 85 °C for 30 min) were removed from their plastic casings and stored overnight at 4 °C (under vacuum packaging), after which the sausages were kept at room temperature for 1 h, followed by sensory evaluation. We held two sensory evaluation sessions, and the same panelists were used in both sessions. All sausages were randomly sampled and each panelist tested samples from all treatments. Before starting the test, each panelist voluntarily signed an informed consent form, ensuring personal data protection and confidentiality. 

The sausages were evaluated in terms of appearance (from “porous fissures” to “compact and smooth”; scale 1–20), color (from “uneven and particles” to “uniform and without obvious particles”; scale 1–20), flavor (from “no aroma” to “rich aroma”; scale 1–20), texture (from “no springiness” to “full springiness”; scale 1–20), and acceptability (from “unacceptable” to “completely acceptable”; scale 1–20) [33], as shown in Table 2. The participants were required to rinse their mouths with purified water before tasting each sample.

### 4.9. Analysis of the Antioxidant Activity of Sausages

To evaluate the antioxidant activity of the sausages, the chopped sausages (4 g) were mixed with distilled water (20 mL) and processed via a digital high-speed homogenization and dispersion machine (FJ300-PSH, Shanghai Specimen Model Factory Co., Ltd., Shanghai, China) at 10,000 r/min for 2 min. Afterward, the mixture was stirred in a water bath at 30 °C for 1 h and filtered to obtain the sample mixture. The TBARS values and DPPH and ABTS free radical scavenging capacities of the sausages were evaluated via the methods of Wang and Xiong [34], Shahabi Mohammadabadi, Goli, and Naji Tabasi [35], and Dai et al. [36], with some modifications. The specific measurement procedures can be found in the Supplementary Materials.

### 4.10. Statistical Analysis

The sausage production and cooking process was replicated three times on different days. To analyze the sensory profiling data, a one-way analysis of variance (ANOVA; with sausages containing different emulsion gels as the only treatment effect) was carried out. The data were processed via IBM SPSS Statistics 22 software and the results were expressed as the means ± standard errors. In addition, the data were subjected to Duncan’s multiple range tests, with a significance level of *p* < 0.05, and Origin 9.0 software Originlab, (Northampton, MA, USA) was used for graphing.

## Figures and Tables

**Table 1:** Textural characteristics of sausages after replacing pork backfat with different protein emulsion gels.

	Hardness (N)	Springiness	Cohesiveness	Resilience
Control	25.84 ± 0.82 ^d^	0.824 ± 0.016 ^e^	0.766 ± 0.005 ^f^	0.421 ± 0.006 ^e^
PPEG-20%	28.57 ± 0.62 ^c^	0.854 ± 0.006 ^cd^	0.767 ± 0.003 ^ef^	0.426 ± 0.004 ^e^
PPEG-40%	29.81 ± 0.58 ^bc^	0.855 ± 0.003 ^cd^	0.775 ± 0.003 ^bcde^	0.444 ± 0.005 ^bcd^
PPEG-100%	40.51 ± 0.48 ^a^	0.881 ± 0.006 ^ab^	0.781 ± 0.001 ^abc^	0.449 ± 0.001 ^abcd^
UPPEG-20%	30.22 ± 0.38 ^bc^	0.854 ± 0.007 ^cd^	0.774 ± 0.001 ^cdef^	0.443 ± 0.003 ^bcd^
UPPEG-40%	30.81 ± 0.55 ^b^	0.860 ± 0.005 ^bcd^	0.774 ± 0.002 ^cdef^	0.439 ± 0.002 ^cd^
UPPEG-100%	41.56 ± 0.41 ^a^	0.884 ± 0.003 ^a^	0.779 ± 0.002 ^abcd^	0.450 ± 0.003 ^abc^
SPEG-20%	28.57 ± 0.65 ^c^	0.840 ± 0.004 ^de^	0.771 ± 0.001 ^def^	0.437 ± 0.002 ^d^
SPEG-40%	30.02 ± 0.86 ^bc^	0.841 ± 0.01 ^de^	0.784 ± 0.004 ^a^	0.453 ± 0.004 ^ab^
SPEG-100%	39.76 ± 0.47 ^a^	0.876 ± 0.003 ^abc^	0.782 ± 0.001 ^ab^	0.456 ± 0.001 ^a^

Control: without emulsion gel; PPEG: peanut protein emulsion gel; UPPEG: ultrasound-modified peanut protein emulsion gel; SPEG: soy protein emulsion gel. Values are mean ± standard error (*n* = 3). Different letters in the same column indicate significant differences (*p* < 0.05).

**Table 2:** Sensory evaluation scores of sausages with different protein emulsion gels.

	Appearance	Color	Flavor	Texture	Acceptability
Control	14.15 ± 0.05 ^a^	15.50 ± 0.90 ^a^	14.85 ± 0.75 ^a^	14.55 ± 1.05 ^b^	14.90 ± 0.50 ^c^
PPEG-40%	16.40 ± 1.00 ^a^	16.00 ± 1.00 ^a^	16.15 ± 0.85 ^a^	16.15 ± 1.05 ^ab^	16.25 ± 0.75 ^abc^
PPEG-100%	16.50 ± 0.90 ^a^	17.15 ± 0.25 ^a^	16.85 ± 0.55 ^a^	17.25 ± 0.75 ^ab^	16.70 ± 0.30 ^abc^
UPPEG-40%	15.20 ± 1.20 ^a^	15.35 ± 0.65 ^a^	15.65 ± 1.35 ^a^	16.20 ± 1.00 ^ab^	15.80 ± 0.80 ^bc^
UPPEG-100%	16.90 ± 0.50 ^a^	17.30 ± 0.50 ^a^	17.00 ± 0.60 ^a^	17.90 ± 0.70 ^a^	18.00 ± 0.20 ^a^
SPEG-40%	14.60 ± 1.00 ^a^	14.80 ± 0.80 ^a^	15.30 ± 0.70 ^a^	15.30 ± 0.70 ^ab^	15.20 ± 0.60 ^bc^
SPEG-100%	16.05 ± 0.75 ^a^	15.90 ± 0.90 ^a^	16.55 ± 1.45 ^a^	16.65 ± 0.55 ^ab^	16.95 ± 0.45 ^ab^

Control: without emulsion gel; PPEG: peanut protein emulsion gel; UPPEG: ultrasound-modified peanut protein emulsion gel; SPEG: soy protein emulsion gel. Values are mean ± standard error (*n* = 15). Different letters in the same column indicate significant differences (*p* < 0.05).

**Table 3:** Sausage formulations.

Ingredients	Backfat Substitution Ratio (%)			
	0	20	40	100
Lean pork (g)	60	60	60	60
Pork backfat (g)	20	16	12	0
Emulsion gel (g)	0	13.34	26	20
Water (g)	18	8.66	0	0
Salt (g)	1.7	1.7	1.7	1.7
Sodium tripolyphosphate (g)	0.3	0.3	0.3	0.3

## Data Availability

The original contributions presented in this study are included in the article material. Further inquiries can be directed to the corresponding author.

## Supplementary Materials

The supporting information can be downloaded at: https://www.mdpi.com/article/10.3390/gels11010062/s1, Method S1: Analysis of the antioxidant activity of sausages.
