# Supplementary material for: Peanut and Soy Protein-Based Emulsion Gels Loaded with Curcumin as a New Fat Substitute in Sausages: A Comparative Study
Source: Gels. 2025 Jan 13;11(1):62. doi: 10.3390/gels11010062 (PMC11765018; doi:10.3390/gels11010062)
Supplement: Supplementary file 1 [file gels-11-00062-s001.zip › gels-3395806-supplementary.pdf]

Fat oxidation was evaluated by determining the content of thiobarbituric acid reactive substances (TBARS) according to the following procedures. The chopped sausage (2 g) was mixed with 8.5 mL of trichloroacetic acid solution (2.5%) and 1.5 mL of TBA (1%) solution and heated at 100 °C in a water bath (HH-2J, Shanghai Weicheng Instrument Co., Ltd.) for 30 min. The supernatant cooled to room temperature was mixed with an equal volume of chloroform and subsequently centrifuged (DT5-4B, Beijing Times Beili Centrifuge Co., Ltd.) at 2000×g for 10 min. The absorbance of the mixture was measured at 532 nm. The TBARS value, expressed as mg malondialdehyde per kg sample (mg MDA/kg), was calculated as follows:

$$\text{TBARS (mg MDA/kg)} = \frac{A_{532}}{M_s} \times 9.48$$

where A_{532} is the absorbance value at 532 nm, M_s is the weight (g) of the sausage sample, and "9.48" is a constant derived from the dilution factor and the molar extinction coefficient (152,000 M^{-1} cm^{-1}) of the red TBA reaction product.

The DPPH radical scavenging capacity of the sausages was evaluated according to the following procedures. One milliliter of mixture or distilled water (as a control) was mixed with 4 mL of DPPH solution (0.1 mM, diluted with 95% methanol) and incubated for 30 min at room temperature in the dark. The absorbance was measured at 517 nm. Similarly, 1 mL of sample mixture was mixed with 4 mL of 95% methanol (as a blank) and incubated for 6 min at room temperature in the dark, after which the absorbance was measured at 734 nm. The DPPH radical scavenging capacity was calculated as follows:

$$\text{DPPH radical scavenging capacity (\%)} = \frac{A_{control} - (A_{sample} - A_{blank})}{A_{control}} \times 100\%$$

The ABTS radical scavenging capacity of the sausages was evaluated according to the following procedures. The mixture was reacted with 10 mL of potassium persulfate (2.45 mmol/L) and 10 mL of ABTS (7 mmol/L) in the dark at room temperature for 12–16 h. The mixture was diluted with 0.1 mM phosphate buffer (pH 7.2) to achieve an absorbance of 0.70 ± 0.02 at 734 nm, and the ABTS working solution was obtained. One hundred microliters of sample solution or distilled water (as a control) was mixed with 4 mL of ABTS working solution and incubated for 6 min at room temperature in the dark. The absorbance was measured at 734 nm. The ABTS radical scavenging capacity was calculated as follows:

$$\text{ABTS radical scavenging capacity (\%)} = \frac{A_{control} - A_{sample}}{A_{control}} \times 100\%$$
